# Development of a Mucoadhesive and In Situ Gelling Formulation Based on κ-Carrageenan for Application on Oral Mucosa and Esophagus Walls. I. A Functional In Vitro Characterization

**DOI:** 10.3390/md17020112

**Published:** 2019-02-12

**Authors:** Barbara Vigani, Angela Faccendini, Silvia Rossi, Giuseppina Sandri, Maria Cristina Bonferoni, Matteo Gentile, Franca Ferrari

**Affiliations:** Department of Drug Sciences, University of Pavia, Viale Taramelli, 12 Pavia, Italy; barbara.vigani@unipv.it (B.V.); angela.faccendini@gmail.com (A.F.); giuseppina.sandri@unipv.it (G.S.); cbonferoni@unipv.it (M.C.B.); matteo.gentile01@universitadipavia.it (M.G.); franca.ferrari@unipv.it (F.F.)

**Keywords:** oral mucositis, κ-carrageenan, polymer–ion interaction, in situ gelation, mucoadhesion, washability test

## Abstract

Oral mucositis and esophagitis represent the most frequent and clinically significant complications of cytoreductive chemotherapy and radiotherapy, which severely compromise the patient quality of life. The local application of polymeric gels could protect the injured tissues, alleviating the most painful symptoms. The present work aims at developing in situ gelling formulations for the treatment of oral mucositis and esophagitis. To reach these targets, κ-carrageenan (κ-CG) was selected as a polymer having wound healing properties and able to gelify in the presence of saliva ions, while hydroxypropyl cellulose (HPC) was used to improve the mucoadhesive properties of the formulations. CaCl_2_ was identified as a salt able to enhance the interaction between κ-CG and saliva ions. Different salt and polymer concentrations were investigated in order to obtain a formulation having the following features: (i) low viscosity at room temperature to facilitate administration, (ii) marked elastic properties at 37 °C, functional to a protective action towards damaged tissues, and (iii) mucoadhesive properties. Prototypes characterized by different κ-CG, HPC, and CaCl_2_ concentrations were subjected to a thorough rheological characterization and to in vitro mucoadhesion and washability tests. The overall results pointed out the ability of the developed formulations to produce a gel able to interact with saliva ions and to adhere to the biological substrates.

## 1. Introduction

Oral mucositis and esophagitis represent the most frequent and clinically significant complications of cytoreductive chemotherapy and radiotherapy. These pathological conditions refer to severe inflamed ulcerative lesions of both the oral mucosa and the esophagus walls, associated with an intense pain [1,2,3]. In the most severe forms, deep ulcerations are also present. Mucositis and esophagitis could impair patient ability to feed and swallow, slowing down the healing process and reducing the resistance to infections. The patient quality of life is severely compromised [4]. The local application of polymeric gels could protect the injured tissues from additional damages, alleviating the most painful symptoms. In particular, they should be characterized by an easy administration and by a prolonged permanence on the damaged tissues. There are two main strategies proposed in the literature to reach such targets. The first one is represented by mucoadhesive formulations, which are able to withstand the physiological removal mechanisms thanks to the formation of physical and chemical bonds with the mucosa. The second strategy, instead, is constituted by gelling liquid systems that undergo a sol–gel transition upon administration, due to their capability to interact with environmental ions or to their sensitivity to temperature changes [5,6,7,8,9]. 

Carrageenans (CGs) are an important class of hydrophilic sulfated marine polysaccharides widely employed as excipients in the pharmaceutical industry [10]. They are mainly obtained by extraction with water or aqueous alkali from some members of the class Rhodophyceae (red seaweed), such as *Chondrus*, *Eucheuma*, *Gigartina*, and *Hypnea* [11,12]. 

CGs are mainly composed of potassium, sodium, calcium, magnesium, and ammonium sulfate esters of galactose and 3,6-anydrogalactose copolymers. These hexoses are alternately linked at the α-1,3 and β-1,4 sites in the polymer. CGs are classified according to the degree of substitution on their free hydroxyl groups. Substitutions are represented by ester sulfate or 3,6-anhydride on the 4-linked residues. Three primary classes of CGs are available, depending on the number and position of the ether sulfate groups, named kappa (κ), iota (ι), and lambda (λ) CGs. κ-CG is a strongly gelling polymer that contains 25% ester sulfate by weight and approximately 34% 3, 6-anhydrogalactose. Gel formation involves a coil-to-helix conformation transition, followed by helix aggregation [13]. This process is thermosensitive and responsive to ions. κ-CG possesses one negative charge per disaccharide unit, which is responsible for the formation of strong and rigid gels. Stronger gels form in presence of K^+^ with respect to Na^+^, Mg^2+^, and Ca^2+^ [13].

Many studies dealt with the use of CGs in addition to other polymers to obtain a controlled drug release, thanks to CG gelling properties. Liu et al. studied the effect of κ-CG on the sustained-release properties of poloxamer 407-based vaginal in situ gel, loaded with acyclovir. In vitro drug release profiles indicated that κ-CG significantly decreased the release rate of acyclovir, retarded the dissolution of poloxamer, and slowed down the gel erosion in a concentration-dependent manner. Moreover, the presence of CG did not impair poloxamer capability to gelify on increasing temperature and significantly prolonged in vivo drug local residence in an animal model. In addition, κ-CG showed a synergistic bioadhesive effect with acrylic acid polymers (Carbopol^®^) [14]. Tomoda et al. developed CG microspheres containing allopurinol and local anesthetic agents for the treatment of oral mucositis [15]. The developed microspheres were able to control drug release and to spread on the surface of the oral cavity.

Li et al. proposed the use of mixtures of κ- and ι-CG in association with potassium chloride for the preparation of vaginal semisolid ovoid microbicide formulations [16].

CGs are generally regarded as relatively non-toxic materials when used in non-parenteral formulations [17]. They show potential pharmaceutical properties, including anticoagulant, anticancer, anti-hyperlipidemic, antiviral, and immunomodulatory activity [10]. Rocha de Souza et al. proved that CGs are characterized by antioxidant and free radical scavenging activity, which makes such polymers good candidates for formulations intended for wound healing [18]. Moreover, studies on the effect of CGs on mucoadhesion to the oropharyngeal area have demonstrated that CGs possess mucoadhesive potential and can be used as enabling excipients in formulations for oral and buccal drug delivery [10,19,20,21]. Lefnaoui et al. prepared buccal gels based on κ-CG and pregelatinized starch blends, containing 2% w/w of miconazole [20]. Other authors developed a buccal matrix containing κ-CG as a mucoadhesive agent for the delivery of pravastatin sodium in the oral cavity. Histological evaluation of porcine buccal mucosa upon contact for 2 h with the formulation proved that it did not cause any tissue damage [21]. 

The present work aims at developing in situ gelling formulations for the treatment of oral mucositis and esophagitis induced by cancer therapies. In particular, the formulations developed, after dosing in a measuring cup, are intended to rinse the oral cavity for 1 min and to be swallowed. κ-CG was selected as a polymer having wound healing properties and able to gelify in presence of saliva ions, while hydroxypropyl cellulose (HPC) was used to improve the mucoadhesive properties of the formulations. CaCl_2_ was identified as a salt able to enhance the interaction between κ-CG and saliva ions. Different salt and polymer concentrations were investigated in order to obtain a formulation having the following features: (i) low viscosity at room temperature to facilitate administration, (ii) marked elastic properties at 37 °C, functional to a protective action towards damaged tissues, and (iii) mucoadhesive properties. 

Prototypes characterized by different κ-CG, HPC, and CaCl_2_ concentrations were subjected to a thorough rheological characterization and to in vitro mucoadhesion tests. In particular, the rheological properties (viscosity and viscoelasticity) of the formulations were investigated at 25 °C to evaluate their resistance to flow during administration, and at 37 °C, after 3:1 w/w dilution in artificial saliva, to evaluate their behavior after administration [22,23]. 

In order to clarify the role of CaCl_2_ as an enhancer of κ-CG/saliva ions interactions, the intrinsic viscosity of κ-CG in presence of different concentrations of CaCl_2_ and its mixture with artificial saliva was studied. 

The mucoadhesive properties were evaluated in vitro by means of a tensile tester, using a commercial mucin suspension as a biological substrate [24]. Moreover, wash away measurements [24] were performed, using porcine esophagus and setting different contact times between the formulations and the tissue.

Since the administration of the formulation could involve a preliminary dilution in water, the prototypes were also characterized after dilution in distilled water, assuming a formulation-to-water dilution ratio equal to 3:1 w/w.

## 2. Results and Discussion

### 2.1. Influence of CaCl_2_ Concentration on κ-CG/Saliva Ions Interaction

**Figure 1:**
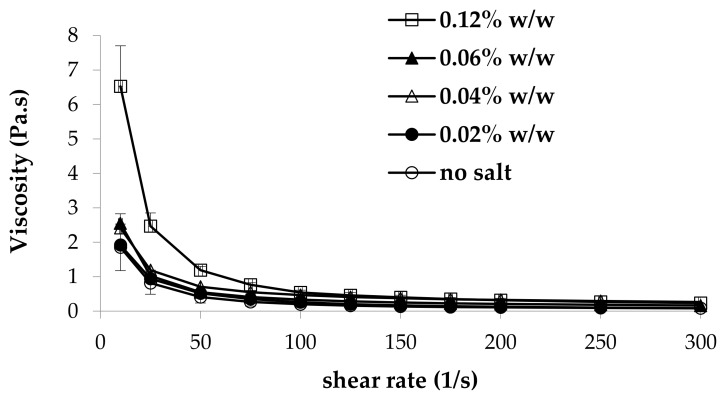
Viscosity curves of 0.8% w/w κ-carrageenan (κ-CG)–1% w/w hydroxypropyl cellulose (HPC) solutions in the presence of increasing CaCl_2_ concentrations (mean values ± s.e.; *n* = 3).

In Figure 1, viscosity curves at 25 °C of 0.8% w/w κ-CG–1% w/w HPC solutions in the presence of increasing concentrations of CaCl_2_ are reported. It can be observed that all the solutions show a shear thinning behavior (i.e., a decrease of viscosity with increasing shear rate). Such behavior is functional to an easy administration: the greater the stress applied, the lower the resistance to flow. It indicates that the samples possess protection/lubricant properties, acting as stress absorbers when small stresses are applied, and as lubricants when subjected to high shear stresses like those involved during administration (pouring from the bottle to the measuring cup and from the measuring cup into the oral cavity) [25].

Moreover, as expected, an increase in CaCl_2_ concentration produces an increase in viscosity. In fact, κ-CG has the ability to form a three-dimensional network in the presence of either monovalent or divalent ions. In particular, calcium ions form gels having the same extent of rigidity as those obtained from monovalent cations. It is known that the concentration of the external counter-ions plays an important role in the κ-CG solution properties, decreasing the effective charge density of the polysaccharide [26,27]. In general, an increase in ion concentration leads to an increase in gel stiffness. This is due to the neutralization of the electrostatic charges of the polysaccharide chains, which enhances their association. 

**Figure 2:**
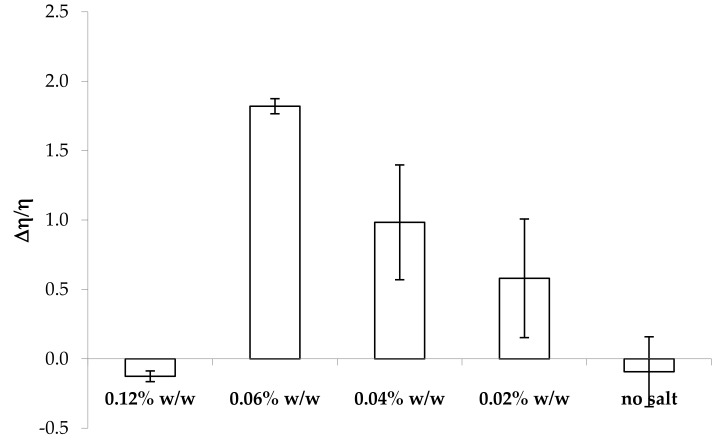
Values of the normalized viscous interaction parameter (Δη/η) obtained for all the samples considered upon 3:1 w/w dilution in artificial saliva (mean values ± s.e.; *n* = 3).

In Figure 2, the values of the normalized viscous interaction parameter (Δη/η) obtained for all the solutions considered upon 3:1 w/w dilution in artificial saliva are reported. Positive values of this parameter indicate an increase in sample viscosity due to the interaction with saliva ions [28]. It can be observed that the presence of CaCl_2_ is necessary for the occurrence of such interactions. Positive values of Δη/η are observed for κ-CG:CaCl_2_ w/w ratios in the range 1:0.25–1:0.075. In absence of CaCl_2_, κ-CG at the concentration of 0.8% w/w does not show any significant changes in viscosity upon dilution in artificial saliva. In fact, the solution without salt is characterized by a Δη/η value not different from zero. On the other hand, excessive amounts of CaCl_2_ are responsible for a missed interaction/gelation. This could be due to a “saturation” of the functional groups responsible for the interaction. This is in line with data reported in literature: in the presence of a high calcium ion concentration, aggregation and precipitation occur, due to a large increase in the number of branches during gelation [29,30]. 

The highest Δη/η value is observed for a CaCl_2_ concentration equal to 0.06% w/w, corresponding to a κ-CG:CaCl_2_ w/w ratio equal to 1:0.075. Moreover, it can be observed that the presence of CaCl_2_ concentrations lower than 0.06% w/w is responsible for Δη/η value characterized by a high variability. This could be due to a poor sample homogeneity after saliva interaction, caused by the fact that the salt amount is not sufficient to induce a complete sample gelation. 

**Figure 3:**
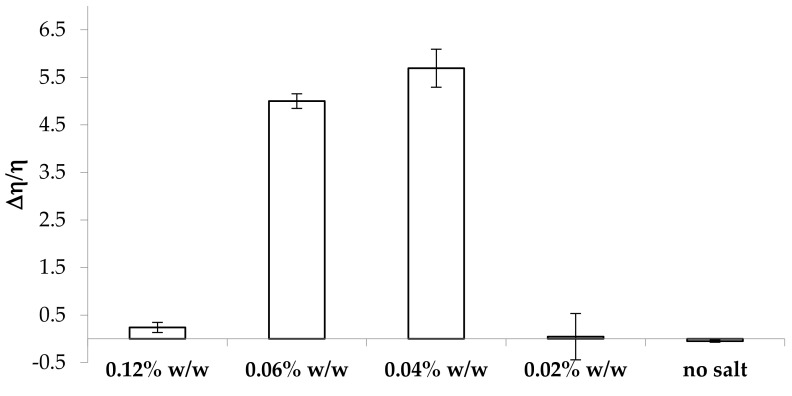
Values of the normalized viscous interaction parameter (Δη/η) obtained for all the samples considered after a double dilution (3:1 w/w in distilled water and 3:1 w/w in artificial saliva) (mean values ± s.e.; *n* = 3).

In order to mimic the eventual dilution of the formulation before administration, all the solutions considered above have been subjected to the same characterization after a first dilution of 3:1 w/w in deionized water and a subsequent dilution of 3:1 w/w in artificial saliva. In Figure 3, the values of the normalized viscous interaction parameter (Δη/η) obtained for all the samples subjected to a double dilution are reported.

The samples containing CaCl_2_ concentrations equal or higher than 0.04% w/w are characterized by positive values of the normalized viscous interaction parameter, indicating an increase in viscosity due to saliva ions also after dilution in water. The highest values of Δη/η are observed for the samples containing 0.04% and 0.06% w/w CaCl_2_ concentrations, corresponding to 0.03% and 0.045% w/w concentrations, respectively, after dilution in water. Considering that κ-CG is subjected to dilution in water, its concentration becomes 0.6% w/w. The highest values of Δη/η are observed for κ-CG:CaCl_2_ w/w ratios equal to 1:0.05 and 1:0.075, confirming the results previously obtained (Figure 2).

In order to explain the results obtained, the intrinsic viscosities, [η], of κ-CG in the presence of different CaCl_2_ concentrations and of artificial saliva were measured.

In Table 1, [η] values of κ-CG in the different solvents investigated are reported.

It is known that polyelectrolytes show a decrease in intrinsic viscosity with increasing ionic strength of the dissolution medium. In deionized water, the intrinsic viscosity value corresponds to a highly expanded coil, due to the fact that polymer chains are not shielded. A progressive decrease of intrinsic viscosity with increasing ionic strength indicates the presence of more compact chains due to the interaction with ions. The intrinsic viscosity value markedly decreases when CaCl_2_ concentration increases to 0.04% w/w, indicating a tendency for aggregation. Intrinsic viscosity reduces by an order of magnitude in artificial saliva (with respect to deionized water), pointing out the occurrence of chain aggregation [25]. 

The results so far obtained indicate that the change in polymer chain conformation occurring in the presence of a low calcium ion concentration (i.e., for κ-CG:CaCl_2_ w/w ratio in the range of 1:0.05–1:0.075) promotes interaction with saliva ions, since polymer chains are not aggregated and are able to interact further with ions. In contrast, higher calcium ion concentrations produce a compaction/aggregation of chains that impairs the interaction with saliva ions. 

### 2.2 Influence of κ-CG Concentration on κ-CG/Saliva Ion Interaction

In order to assess the influence of κ-CG concentration on the polymer capability to interact with saliva ions, different κ-CG concentrations (0.8%, 0.6%, 0.4%, and 0.2% w/w) were considered, while maintaining fixed CaCl_2_ (0.04% w/w) and HPC (1% w/w). 

**Figure 4:**
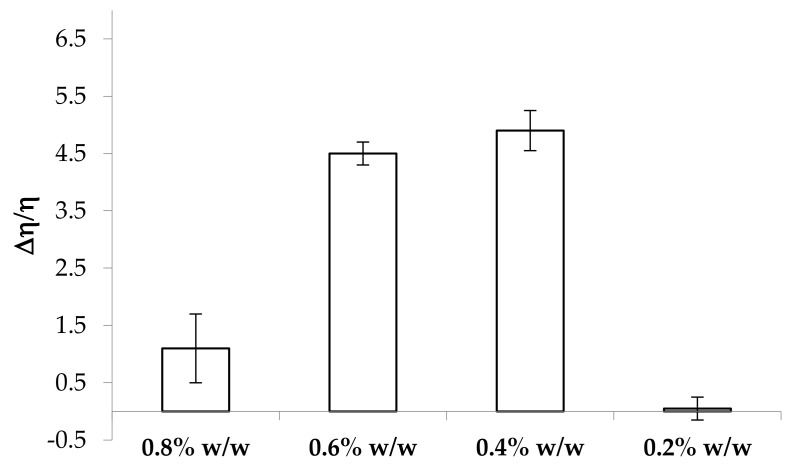
Values of the normalized viscous interaction parameter (Δη/η) obtained for the samples containing increasing κ-CG concentrations after 3:1 w/w dilution in artificial saliva (mean values ± s.e.; *n* = 3).

In Figure 4, the values of the normalized viscous interaction parameter (Δη/η) obtained for all the solutions considered upon dilution in artificial saliva are reported. It can be observed that a decrease of polymer concentration of up to 0.4% w/w produces an increase in κ-CG capability to interact with saliva ions. Positive values of the interaction parameter are obtained for 0.8%, 0.6%, and 0.4% w/w κ-CG concentrations in the presence of 0.04% w/w CaCl_2_ concentration, corresponding to κ-CG:CaCl_2_ w/w ratios in the range of 1:0.1–1:0.05. A value of Δη/η not significantly different from 0 is observed for 0.2% w/w κ-CG concentration, corresponding to a 1:0.2 κ-CG:CaCl_2_ w/w ratio. This indicates the lack of an interaction with saliva ions. 

### 2.3 Influence of HPC Concentration on Mucoadhesive Properties

**Figure 5:**
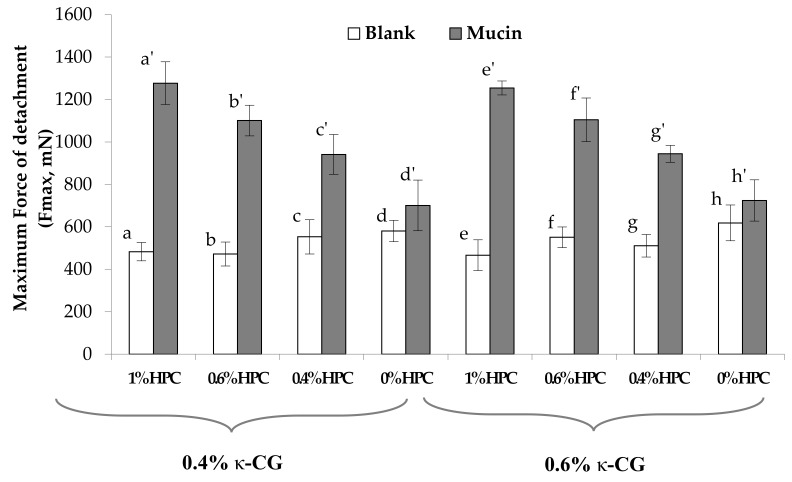
Values of the maximum force of detachment, observed for κ-CG solutions containing different HPC concentrations. Blank: measurement performed in absence of mucin. In all the samples, 0.04% w/w CaCl_2_ was present (mean values ± s.e.; *n* = 6). Anova one way, Multiple Range Test (*p* < 0.05): a vs a’; b vs b’; c vs c’; e vs e’; f vs f’; g vs g’.

Figure 5 reports the values of the maximum force of detachment (Fmax) observed for the solutions containing 0.4% and 0.6% w/w κ-CG, increasing concentrations of HPC, and 0.04% w/w CaCl_2_ in the presence of artificial saliva. No statistically significant differences are observed between Fmax values obtained in presence and absence (blank) of mucin for the solution without HPC. This indicates the absence of mucoadhesive properties [28]. All the solutions containing HPC are able to interact with the biological substrate, presenting Fmax values in the presence of mucin significantly higher than those obtained from blank measurements.

**Figure 6:**
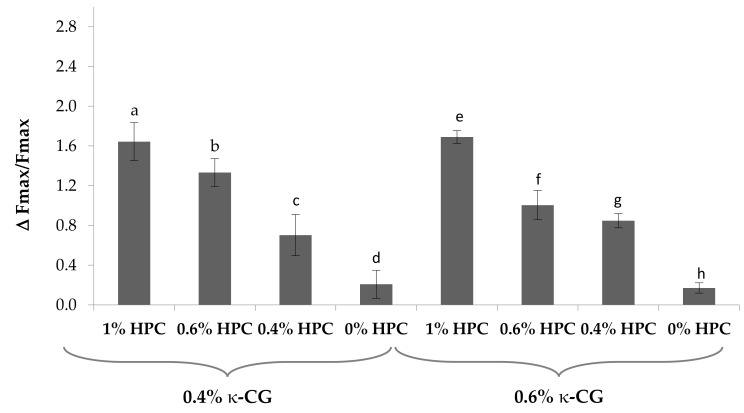
Values of the normalized mucoadhesion parameter (ΔFmax/Fmax), observed for κ-CG solutions containing different HPC concentrations. In all the samples, 0.04% w/w CaCl_2_ was present (mean values ± s.e.; *n* = 6). Anova one way, Multiple Range Test (*p* < 0.05): a vs c, d; b vs c, d; c vs d; e vs f, g; f vs h; g vs h.

To compare the performances of the different solutions on a homogeneous basis, the normalized mucoadhesion interaction parameter (ΔFmax/Fmax) was calculated for each of them. Figure 6 reports the values of this parameter for all the samples under examination. It can be observed that an increase in HPC concentration from 0.4% to 1% w/w produces a significant increase in the mucoadhesion potential, indicated by an increase in the ΔFmax/Fmax value.

On the basis of the results so far obtained, it was decided to continue the study on the formulations containing 0.4% and 0.6% w/w κ-CG and 0.6% and 1% w/w HPC.

### 2.4. Rheological Characterization of κ-CG (0.4%–0.6% w/w)/HPC (0.6%–1% w/w)/CaCl_2_ (0.04% w/w) Solutions

**Figure 7:**
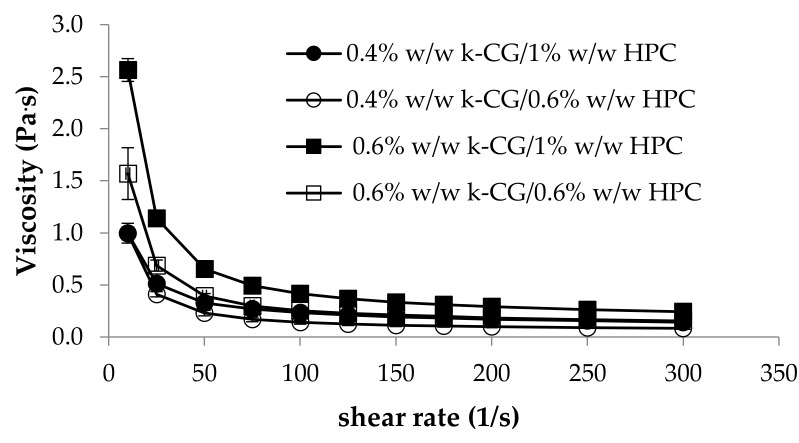
Viscosity profiles of κ-CG (0.4%–0.6% w/w)/HPC (0.6–1% w/w)/CaCl_2_ (0.04% w/w) solutions at 25 °C (mean values ± s.e.; *n* = 3).

In Figure 7, the viscosity curves of κ-CG (0.4%–0.6% w/w)/HPC (0.6%–1% w/w)/CaCl_2_ (0.04% w/w) solutions are compared at 25 °C. It can be observed that at high shear rates (>100 s^-1^), 0.4% w/w κ-CG–1% w/w HPC and 0.6% w/w κ-CG–0.6% w/w HPC solutions show comparable viscosity values, meaning that they are characterized by the same resistance to flow during administration. As expected, at all the shear rates considered, the highest viscosity is shown by the solution containing the higher concentrations of both polymers (0.6% w/w κ-CG–1% w/w HPC). All the solutions are characterized by a shear thinning behavior as indicated by the decrease of viscosity with increasing shear rate. Such a behavior implies that the solution is more viscous when small stresses are applied, acting as stress absorber, while it is less viscous, opposing less resistance, when subjected to high shear stresses, as during administration [25].

**Figure 8:**
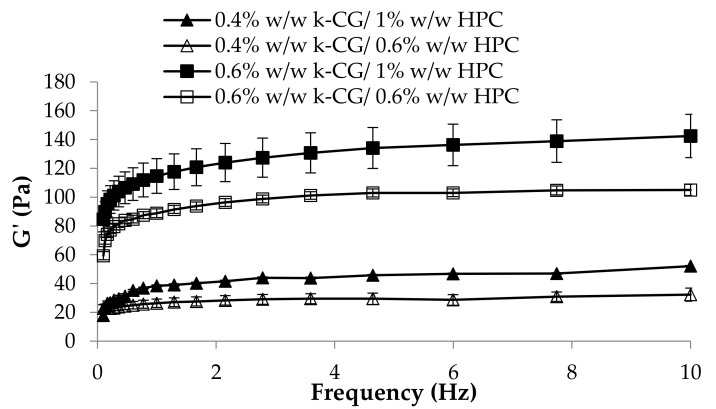
G’ (storage elastic modulus) of κ-CG (0.4%–0.6% w/w)/HPC (0.6%–1% w/w)/CaCl_2_ (0.04% w/w) solutions at 25°C (mean ± s.e.; *n* = 3).

As for the elastic properties, expressed by the storage elastic modulus G’, the main important role is played by κ-CG: the solutions containing 0.6% w/w κ-CG are characterized by G’ profiles higher than those of the solutions based on 0.4% w/w κ-CG (Figure 8). Low elastic properties at 25°C are preferable, since a sample characterized by a prevalence of the viscous properties on the elastic ones flows more easily from the containers (bottle and measuring cup).

**Figure 9:**
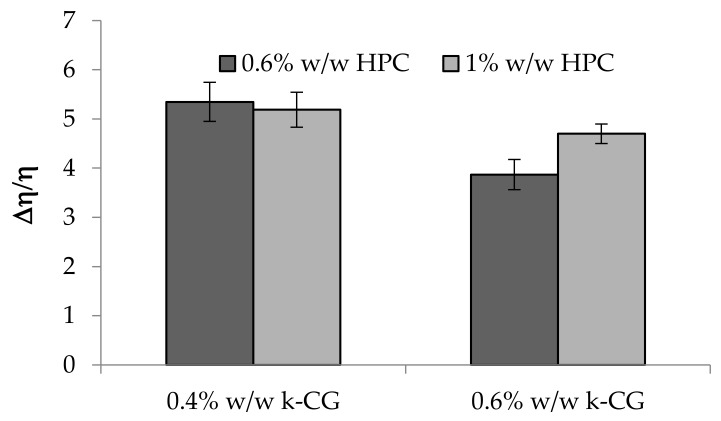
Values of the normalized viscous interaction parameter (Δη/η) of κ-CG (0.4%–0.6% w/w)/HPC (0.6%–1% w/w)/CaCl_2_ (0.04% w/w) solutions upon 3: 1 w/w dilution in artificial saliva (mean ± s.e.; *n* = 3).

In Figure 9, the values of the normalized viscous interaction parameter (Δη/η) of the above mentioned solutions upon dilution in artificial saliva are compared. It can be observed that the increase in HPC concentration from 0.6 to 1% w/w does not produce any significant change in the κ-CG capability to interact with saliva ions. Moreover, such formulations, independently of HPC concentration, are able to interact with saliva ions in a very short time. In fact, they show an increase in viscosity as early as after 1 min contact with saliva ions. As an example, for 0.4% w/w κ–CG/1% w/w HPC, viscosity values at low shear rates equal to 0.83 Pa.s and 0.40 Pa.s are observed upon 1 min contact with artificial saliva (S) and distilled water (3:1 weight ratio), respectively.

**Figure 10:**
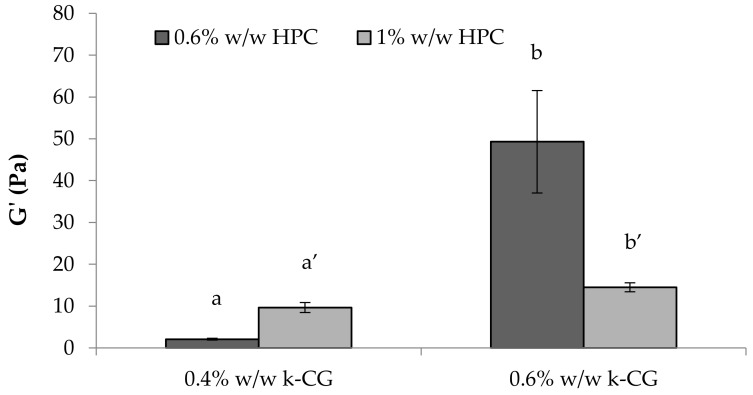
G’ (storage elastic modulus) values of κ-CG (0.4%–0.6% w/w)/HPC (0.6%–1% w/w)/CaCl_2_ (0.04% w/w) solutions upon 3:1 w/w dilution in artificial saliva (mean ± s.e.; *n* = 3). Anova one way, Multiple Range Test (*p* < 0.05): a vs a’; b vs b’.

In Figure 10, G’ values of κ-CG (0.4%–0.6% w/w)/HPC (0.6%–1% w/w)/CaCl_2_ (0.04% w/w) solutions upon dilution in artificial saliva are compared. The presence of a high CG concentration is responsible for a greater strengthening of the polymer solution after saliva ion interaction, as indicated by the increase in G’ values (Figure 10). This behavior, particularly evident in the presence of 0.6% w/w HPC concentration, is functional to a protective action of the solution towards the application site [24]. The increase in HPC concentration produces an increase in elasticity only in the presence of 0.4% w/w κ-CG. On the contrary, HPC at 1% w/w in the presence of the higher κ-CG concentration is responsible for a weakening of the solution elasticity. This can be attributed to a disturbing effect of HPC, when present at a high concentration, towards the polymer network formed as a response of κ-CG–ion interactions. The higher κ-CG concentration results, upon ion interaction, in a polymer network characterized by a high elasticity. The presence of a high HPC concentration weakens this structure. 

**Figure 11:**
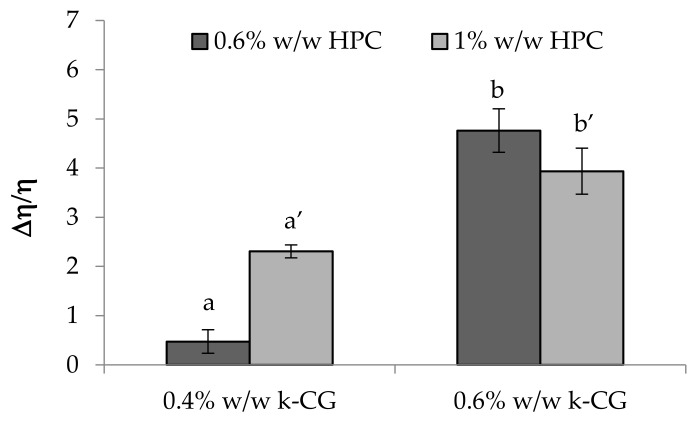
Values of the normalized interaction parameter (Δη/η) obtained for all the samples considered after a double dilution (3: 1 w/w in distilled water and 3:1 w/w in artificial saliva) (mean values ± s.e.; *n* = 3). Anova one way, Multiple Range Test (*p* < 0.05): a vs a’.

In Figure 11, the values of the normalized viscous interaction parameter (Δη/η) obtained for all the samples subjected to a double dilution, in water and in artificial saliva, are reported. As expected, κ-CG, when present at the higher concentration (0.6% w/w), is characterized by the greatest capability to interact with saliva ions. Such ability is not affected by HPC concentration. Upon dilution in water, the actual κ-CG and HPC concentrations are equal to 0.45% w/w and 0.75% w/w, respectively. Such results are in line with those reported in Figure 9. On the contrary, for the 0.4% w/w κ-CG solution (0.3% w/w upon dilution in water), an increase in HPC concentration produces a greater increase in solution viscosity. 

**Figure 12:**
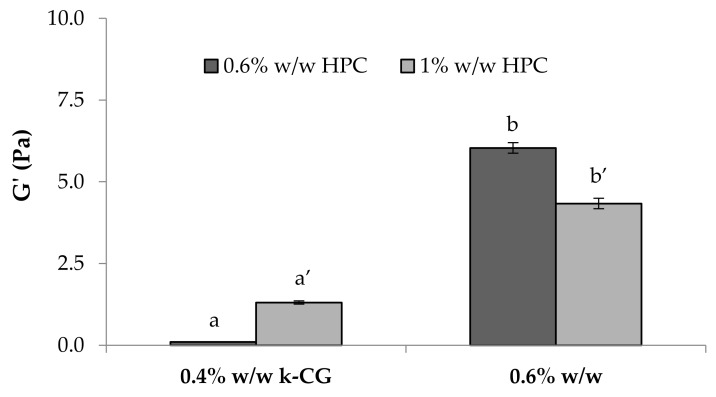
G’ (storage elastic modulus) values of κ-CG (0.4%–0.6% w/w)/HPC (0.6%–1% w/w)/CaCl_2_ (0.04% w/w) solutions after a double dilution (3:1 w/w in distilled water and 3:1 w/w in artificial saliva) (mean values ± s.e.; *n* = 3). Anova one way, Multiple Range Test (*p* < 0.05): a vs a’; b vs b’.

Figure 12 reports the results of the viscoelastic tests performed on the solutions after a double dilution. These confirm that HPC affects in a different way the viscoelastic properties of the solution, depending on its concentration. 

### 2.5. In Vitro Washability Test

**Figure 13:**
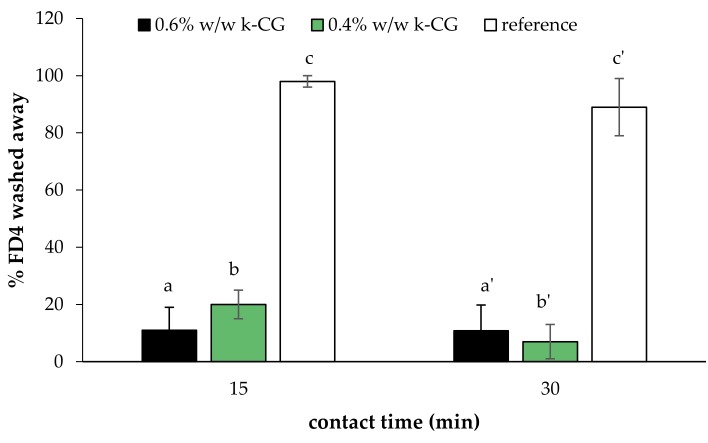
Percentage of the fluorescent probe (%FD4) washed away from esophagus walls after 15 and 30 min contact time (mean values ± s.e.; *n* = 6). Anova one way, multiple range test (*p* < 0.05): a vs c, b vs c; a’ vs c’; b’ vs c’.

Figure 13 shows the results of the washability test performed on κ-CG (0.4%–0.6% w/w)/HPC (1% w/w)/ CaCl_2_ (0.04% w/w) solutions. In particular, the percentage of the fluorescent probe (FD4) washed away from the esophagus wall after 15 and 30 min contact time is reported. An aqueous FD4 solution, containing the same FD4 amount as polymer solutions, was employed as a non-mucoadhesive reference. It can be observed that both polymer solutions are characterized by %FD4 washed away values significantly lower with respect to the reference. This indicates their capability to interact with the biological substrate. No significant differences are observed between the performances of the two solutions.

## 3. Materials and Methods

### 3.1. Materials

κ-Carrageenan (κ-CG), porcine gastric mucin type II, fluorescein isothiocyanate-dextran (average molecular weight = 4000 Da, FD4), and calcium chloride (CaCl_2_) were purchased from Sigma-Aldrich (Milan, Italy). Potassium chloride (KCl), sodium chloride (NaCl), sodium bicarbonate (NaHCO_3_), and sodium phosphate monobasic (NaH_2_PO_4_·H_2_O) were purchased from Carlo Erba Reagents (Milan, Italy). Klucel^™^ hydroxypropylcellulose (HPC) was purchased from Ashland (Schaffhausen, Switzerland).

### 3.2 Preparation of Artificial Saliva

Artificial saliva was prepared dissolving KCl 1.5%, NaCl 0.43%, CaCl_2_ 0.22%, NaHCO_3_ 0.42%, and NaH_2_PO_4_·H_2_O w/v in distilled water [5]. 

### 3.3 Preparation of κ-CG/HPC/CaCl_2_ Solutions

In the present work, solutions based on κ-CG, HPC, and CaCl_2_ at different concentrations (% w/w) were prepared as detailed below. 

Briefly, κ-CG was dissolved in distilled water at 80 °C under mild magnetic stirring (150 rpm). Once κ-CG was solubilized, HPC was added and then maintained under mild magnetic stirring for 2 h at room temperature. Finally, CaCl_2_ was added to κ-CG/HPC mixture that was maintained under mild stirring until complete salt dissolution.

The experimental work was articulated in three phases. In the first phase, CaCl_2_ concentration was varied (0.02%–0.12% w/w) while maintaining constant κ-CG (0.8% w/w) and HPC (1% w/w) concentrations, in order to identify the minimum CaCl_2_ concentration necessary to promote κ-CG gelation after 3:1 (w/w) dilution in artificial saliva. In the second phase, at fixed CaCl_2_ (0.4% w/w) and HPC (1% w/w) concentrations, different κ-CG concentrations were considered (0.8%, 0.6%, and 0.4% w/w) in order to identify the κ-CG:CaCl_2_ ratio, corresponding to optimal in situ gelling properties. In the third phase, different HPC concentrations (1%, 0.6%, and 0.4% w/w) were added to the most promising κ-CG/HPC blends to evaluate the minimum HPC concentration necessary to guarantee the mucoadhesive properties, without altering the in situ gelling behavior.

Prior to each analysis, all polymeric solutions were maintained at rest for 24 h at 4 °C. Such temperature has been chosen to standardize the sample conditions prior analysis.

### 3.4. Viscosity Measurements

Rheological analyses were carried out by means of a rotational rheometer (MCR 102, Anton Paar, I) equipped with a cone plate combination (CP50-1, diameter = 50 mm; angle = 1°) as the measuring system. Viscosity measurements were performed at increasing shear rates in the range 1–300 s^−1^ at 25 °C on the sample as such and at 37 °C on the sample diluted 3:1 w/w in artificial saliva (S) or in distilled water (W), upon 15 min of mild stirring. Such time was chosen to ensure sample homogeneity. The normalized viscous interaction parameter Δη/η was calculated at 50 s^−1^, according to Equation (1):

Δη/η = (η_S_ – η_W_)/η_W_ (1)

where η_S_ is the viscosity measured at 37 °C upon dilution 3:1 w/w in artificial saliva and η_W_ is the viscosity measured at 37 °C upon dilution 3:1 w/w in distilled water. Three replicates were considered for each sample. 

In order to evaluate the gelation time, samples were placed in contact under mild stirring with S (3:1 weight ratio) for 1 min. The viscosity of the sample was measured at 37 °C. The same analysis was performed upon contact with distilled water (3:1 weight ratio). The time of 1 min was chosen since it is the time assumed for the oral rinsing. An increase in viscosity was considered as an index of gelation. 

In order to simulate an eventual dilution of the formulation before administration, viscosity measurements were performed at 37 °C also on the sample subjected to a double dilution (3:1 w/w in distilled water and 3:1 w/w in artificial saliva) and the parameter Δη/η was calculated.

Three replicates were considered for each sample.

### 3.5. Intrinsic Viscosity Measurements

The intrinsic viscosity [η] of κ-CG was evaluated after κ-CG dissolution in different solvents:

- deionized water;
- 0.02% w/w CaCl_2_ in deionized water;
- 0.04% w/w CaCl_2_ in deionized water;
- 0.12% w/w CaCl_2_ in deionized water;
- Artificial saliva.

In detail, 0.3% w/w κ-CG was solubilized in each solvent at 80 °C under magnetic stirring (150 rpm) for 24 h. Each κ-CG solution was gradually diluted in order to obtain the following concentrations: 0.25%, 0.2%, 0.15%, and 0.125% w/w for the samples prepared in deionized water with and without CaCl_2_, and 0.2%, 0.1%, 0.05%, 0.025%, and 0.0125% w/w for the samples dissolved in artificial saliva. 

All the measurements were carried out by means of a capillary viscosimeter Cannon-Fenske PSL ASTM-IP 150 (viscosimeter constant (k) equal to 0.03658 mm^2^/s^2^ at 40°C), which was clamped vertically in a water bath at the constant temperature of 37 °C. Before each analysis, a volume of pure solvent (blank measurement) or polymer solution (polymer dissolved in pure solvent) was introduced into the viscosimeter and maintained at rest for 10 minutes, in order to reach the required temperature. Six replicates were considered for each sample.

For each sample, the dynamic viscosity was calculated according to the Equation (2):

η = kρt(2)

where k was the viscosimeter constant, ρ was the density of the solvent, and t was the time of flow through the viscosimeter capillary of the sample. 

Thereafter, the relative viscosity (η_rel_) was calculated according to the Equation (3):

η_rel_ = η/η_0_ (3)

where η and η_0_ were the viscosities of the polymeric solution and the pure solvent, respectively.

Considering Equation (2), the Equation (3) can be written as follows:

η_rel_ = t/t_0_ (4)

where t and t_0_ were the time of flow through the capillary recorded for the polymer solution and the pure solvent, respectively.

The specific viscosity (η_sp_) and the reduced viscosity (η_red_) were then calculated according to the Equations (5) and (6):

η_sp_ = η_rel_ − 1 (5)

η_red_ = η_sp_/c (6)

where c was the concentration of the polymeric solution.

η_red_ was plotted versus the corresponding polymer concentration (g/dL). The intercept of the straight line that fitted the experimental data corresponds to the intrinsic viscosity [η] value.

### 3.6. Mucoadhesion Measurements

The mucoadhesive properties of κ-CG/HPC/CaCl_2_ solutions, after 3:1 w/w dilution in artificial saliva, were assessed at 37 °C by means of a TA.XT plus Texture Analyzer (Stable Micro Systems, Godalming, UK), equipped with a 1 kg load cell and a cylindrical movable probe (P/10C). A porcine gastric mucin dispersion (8% w/w) was prepared in artificial saliva as a biological substrate. 

Each sample (30 µL) was layered on a filter paper disc (Ø = 10 mm) and fixed on the movable probe. 30 µL of mucin dispersion were fixed, faced to the solution, on the sample holder. A preload of 2500 mN was applied for 300 s. The probe was then lowered to put in contact the mucin dispersion and the diluted sample. The probe was then raised at a constant speed (2.5 mm/s) up to the complete mucin sample separation. Blank measurements were also carried out using 30 µL of artificial saliva instead of the mucin dispersion. Six replicates were considered for each sample.

The maximum detachment force (Fmax, mN) was measured and the normalized differential parameter ΔFmax/Fmax was calculated according to the following equation:

ΔFmax = (Fmax_mucin_ − Fmax_blank_)/ Fmax_blank_ (7)

where Fmax_mucin_ was the maximum force measured in the presence of mucin and Fmax_blank_ was the maximum force measured in the absence of mucin (blank measurements).

### 3.7. Viscoelastic Measurements

Rheological analyses were performed by means of a rotational rheometer (MCR102, Anton Paar, Turin, I), using a C50-1 cone (∅ = 50 mm and ϑ = 1°) as the measuring system.

Sample viscoelasticity was assessed by dynamic oscillatory measurements, such as the stress sweep test and oscillation test. In the stress sweep test, increasing stresses were applied at a constant frequency (0.1 Hz) and the elastic response of the sample, expressed as storage modulus G′, was measured. Such a test allows us to identify the “linear viscoelastic region”. In the oscillation test, a shear stress, chosen in the linear viscoelastic region previously determined, was applied at increasing frequencies (0.1 to 10 Hz) and G’ and G’’ (loss modulus) profiles were recorded. Measurements were performed at 25 °C on κ-CG/HPC/CaCl_2_ solutions as such and at 37 °C on solutions upon dilution 3:1 w/w in artificial saliva. Three replicates were considered for each sample.

### 3.8. In Vitro Washability Test

Finally, a washability test was performed on the most promising κ-CG/HPC/CaCl_2_ solutions using porcine esophagus as the biological substrate. Fluorescein isothiocyanate (FD4), a fluorescent labeling molecule, was added to the polymer solutions in a concentration equal to 0.06% w/w. FD4 aqueous solution (0.06% w/w) was used as reference. Briefly, 50 mg of solution, loaded with FD4 and diluted 3:1 (w/w) in artificial saliva, were put in contact with the substrate at 37 °C for 15 and 30 min. Distilled water was then fluxed for 1 min over the solutions to simulate the removal action of physiologic fluids. Distilled water was collected and subjected to spectrofluorimetric analysis (LS50B, Perkin Elmer) at excitation and emission wavelengths of 490 and 515 nm, respectively.

### 3.9. Statistical Analysis

Whenever possible, experimental values of the various types of measures were subjected to statistical analysis, carried out by means of the statistical package Statgraphics 5.0 (Statistical Graphics Corporation, Rockville, MD, USA). In particular, one-way ANOVA–Multiple Range Test was used.

## 4. Conclusions

The assessment of the rheological properties of 0.8% w/w κ-CG solutions containing decreasing concentrations of CaCl_2_ led to the choice of a salt concentration equal to 0.04% w/w. The presence of this concentration was responsible for a high value of the normalized interaction parameter Δη/η, indicating a great interaction with saliva ions, as well as for low viscosity values at room temperature, compatible with an easy administration. 

The rheological properties of the solutions containing a fixed CaCl_2_ concentration (0.04% w/w) and decreasing κ-CG amounts indicate the following polymer concentrations as optimal: 0.6% w/w and 0.4% w/w. The change in polymer chain conformation occurring in the presence of a low calcium ion concentration improves the interaction with saliva ions, since polymer chains are not aggregated and are able to interact further with ions. In the presence of a high salt concentration, chains are compacted/aggregated and are unable to interact with saliva ions. The optimal weight ratio between κ-CG and CaCl_2_ to enhance the interaction with saliva ions is in the range 1:0.1–1:0.05. 

The addition of HPC gives mucoadhesive properties to κ-CG/CaCl_2_ solutions without impairing κ-CG capability to interact with saliva ions.

The results of washability studies using porcine esophagus mucosa confirmed the ability of the developed formulations to produce a gel able to interact with saliva ions and to adhere to the biological substrate. The overall results indicate that such formulations represent promising vehicles for the treatment of oral mucositis and esophagitis caused by radio- and chemotherapy.

## Figures and Tables

**Table 1:** Intrinsic viscosity [η] values of κ-CG in different solvents.

Solvent	[η] (dL/g)*
Deionized water	61.1
0.02% w/w CaCl_2_ in water	52.4
0.04% w/w CaCl_2_ in water	12.1
0.12% w/w CaCl_2_ in water	10.6
Artificial saliva	4.8

* mean values (*n* = 6); CV (coefficient of variation)% < 10%.
